# Miltefosine Rescue Treatment for Visceral Leishmaniasis Relapse Patient

**DOI:** 10.1155/2019/3634568

**Published:** 2019-09-03

**Authors:** Anup Bastola, Mitesh Shrestha, Bimal Sharma Chalise, Abhdesh Kumar Mishra, Lina Devkota

**Affiliations:** ^1^Sukraraj Tropical & Infectious Disease Hospital, Kathmandu, Nepal; ^2^Research Institute for Bioscience and Biotechnology, Kathmandu, Nepal

## Abstract

We report the first case of miltefosine rescue treatment carried out for visceral leishmaniasis (VL) relapse patient. Despite undergoing treatment regimens of liposomal amphotericin B (LAMB) 5 mg/kg (standard: 10 mg/kg) daily for 3 days during the first episode followed by LAMB 5 mg/kg stat and paromomycin 15 mg/kg IM for 10 days against the second episode, the patient suffered from a third relapse and was eventually treated with miltefosine 50 mg BID directly observed treatment for 28 days. Prior to treatment, the patient had a history of intermittent fever and vague abdominal pain for one week and epistaxis for 4 days. He had massive splenomegaly, tested positive for the rK39 test, and showed LD bodies in bone marrow aspirate. The patient tested negative for malaria and HIV. Upon treatment completion with miltefosine, the patient had clinically improved and showed no LD bodies in bone marrow.

## 1. Introduction

Visceral leishmaniasis (VL) is one of the endemic neglected tropical diseases in Nepal. The government aims to decrease the indigenous cases of VL to a level that would no longer pose a public health problem. Early detection, active case surveillance, integrated vector management, and effective treatment with best effective medicines available are some of the tools being implemented by the government. As per the national guidelines adopted by the Epidemiology and Disease Control Division, the treatment of VL includes a single dose of liposomal amphotericin B (LAMB) (10 mg/kg) (World Health Organization prequalified AmBisome) or combination therapies of LAMB with paromomycin or miltefosine [1]. LAMB has been shown to be effective with a single intravenous dose of 10 mg/kg with a 98% cure rate [2]. A single dose of LAMB (5 mg/kg) combined with paromomycin (11 mg/kg intramuscular) followed for 10 days gave a cure rate of 98.7% [3]. Here, we report a case from the hilly nonprogrammed district of Nepal who had two relapses following standard treatment.

## 2. Case Presentation

**Figure 1:**
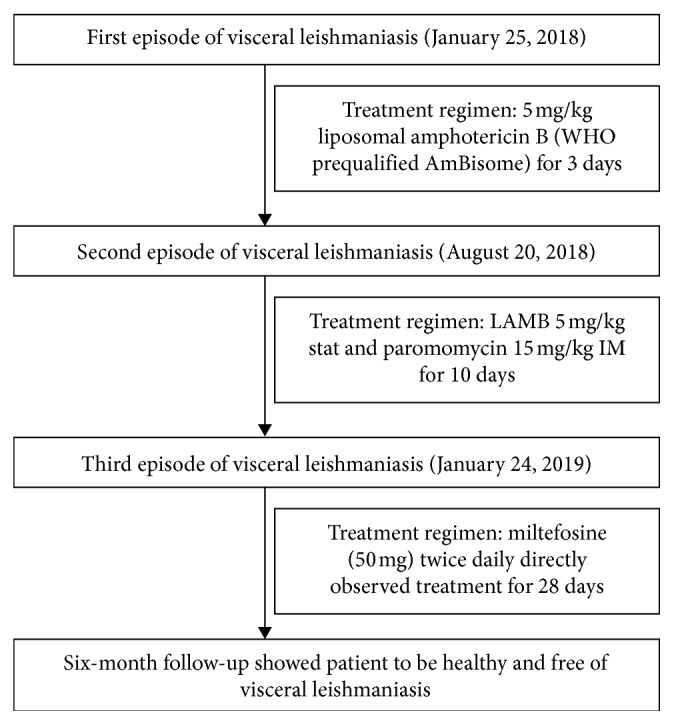
Flow chart of patient case history.

A forty-five-year-old male resident of Saile Sikhar Municipality, Ward no. 5, Darchula District, was admitted to Sukraraj Tropical and Infectious Disease Hospital on 24 January 2019. Prior to visitation to our center, the patient had already been treated with LAMB (5 mg/kg daily for 3 days) against the first episode while LAMB 5 mg/kg stat and paromomycin 15 mg/kg IM for 10 days against the second episode. Finally, the patient was treated with miltefosine (50 mg) twice daily directly observed treatment for 28 days (Figure 1).

## 3. First Episode

The patient presented to a local hospital in Nepalgunj on 25 January 2018 with a history of abdominal pain, anorexia, and fever with chills and sweating for 3 months. Clinically, the patient showed pallor with hepatosplenomegaly. The following information was available in his case file: his rK39 test was positive; glucose level 109.8; urea 17.64; creatinine 0.75; total bilirubin 62; direct B 0.28 mg%; alanine aminotransferase 20.3 U/L; aspartate aminotransferase 21.5 U/L; and alkaline phosphatase 118.6 U/L. He was admitted and treated with three doses of LAMB, 5 mg/kg daily for 3 days in the hospital.

## 4. Second Episode

The patient went to the Zonal Hospital of Western Development Region on 20 August 2018. Bone marrow aspiration cytology was performed showing cellularity which was normal for age; myeloid : erythroid ratio was 1 : 2. There was normal maturation and morphology of normoblasts. Myeloblasts were not identified in the smears studied. The megakaryocyte was normal in morphology and distribution. There was an increase in plasma cells and macrophages. Rare histiocytes showed intracellular amastigotes (LD bodies). Sparse clusters and singly dispersed extracellular amastigotes (LD bodies) were also identified. His total blood count was 1630; neutrophils 11.1%; lymphocytes 68.4%; monocytes 8.9%; eosinophils 0.9%; basophils 10.7%; hemoglobin level 6.8 g/dl; platelet count 104,000; creatinine 0.8 mg/dl; glucose 108.4 mg/dl; alanine aminotransferase 13.8 U/L; urea 30.4.

At the time of admission, the patient had had a history of fever and loss of appetite for 4 weeks, vomiting for 2 weeks, constipation for 4 days, productive cough for 4 days, and bilateral pitting pedal and chest pain for 4 days. Laboratory parameters revealed a hemoglobin level of 5.4 g/dl; total count 800; platelet count 54,000; prothrombin time 10 sec with international normalized ratio 0.91; rK39-positive; malaria- and leptospira-negative; urea and creatinine 20/0.4 mg/dl; total bilirubin 0.7 mg/dl; direct bilirubin 0.02 mg/dl; HIV serology negative; acid-fast bacilli-negative sputum; serum albumin 1.1 mg/dl; and total protein 6.9 mg/dl; urine routine microscopy showed 18–20 pus cells and 8–10 red blood cells. The patient was treated with ceftriaxone, blood transfusion (2 pints), and LAMB 5 mg/kg single dose in day one followed by paromomycin 15 mg/kg IM for 10 days. On the basis of X-ray findings, antitubercular treatment category 1 was also started. Hospital stay was uneventful with no fever, and vitals were stable.

After a week of treatment, his laboratory parameters improved with hemoglobin 8.4 g/dl; total count 2000; platelet count 131,000; and creatinine 0.2 mg/dl. The liver function test showed alanine aminotransferase 24 U/L; aspartate aminotransferase 21 Units/L; total bilirubin 0.6 mg/dl; and direct bilirubin 0.1 mg/dl. Blood transfusion was performed on August 28 and 29. He received 3 units of blood in the hospital and was referred to our center.

## 5. Third Episode

Upon arrival, the patient complained about an intermittent fever and vague abdominal pain for the past one week. He also had epistaxis for 4 days. Clinically, he looked emaciated, pale, lean, and thin. There was massive splenomegaly crossing the midline extending below the umbilicus up to the right iliac fossa. Vitals were within normal limits (blood pressure 90/60 mm of Hg, temperature 96.2°F, and pulse 84/min). Laboratory parameters revealed a hemoglobin level of 5.8 g/dl; total count 400 cells/cumm; neutrophils 69%; lymphocytes 27%; monocytes 4%; and platelet count 29,000 cells/cumm. Peripheral blood smear showed anisopoikilocytosis; hypochromic red blood cells; very low white blood cell count with normal morphology; no abnormal cells; and very low platelet count with normal morphology. rK39 was positive. Sputum for acid-fast bacilli was negative. Lipid profile showed a cholesterol level of 67 mg/dl; triglyceride 136 mg/dl; high-density lipoprotein 20 mg/dl; and low-density lipoprotein 20 mg/dl. Serum albumin was 1.0 mg/dl; total protein 7.9; prothrombin time 15 sec; and international normalized ratio 1.3. Sputum for GeneXpert MTB/RIF was negative. Ferritin level was increased to 766 ng/ml. Bone marrow cytology was performed which showed parasitic Leishman-Donovan (LD) bodies. Chest X-ray was normal. Ultrasonography revealed massive splenomegaly measuring 26 cm. There was hepatomegaly with coarse echotexture. Portal vein caliber distended with mild portal hypertension. There was no lymphadenopathy with minimal ascites. Malarial parasite and HIV serology were negative.

The patient was treated with miltefosine 50 mg twice daily directly observed treatment for 28 days in hospital. The patient's general condition improved with 3 kg weight gain within a month. The spleen regressed and could be felt left to the midline six fingers below the costal margin. His total count improved and increased up to 2000 cells/cumm while the platelet count increased to 190000 cells/cumm. Liver function tests and renal function tests were normal, and bone marrow reports showed markedly hypercellular marrow showing adequate iron stores and no abnormal cells. The LD bodies were not visible in the bone marrow 30 days after treatment with miltefosine.

First follow-up after miltefosine treatment was done six weeks after discharge. The patient had 7 kg weight gain. His blood parameters showed a hemoglobin count of 9.6 gm/dl, total count 3200 cells/cumm, platelet count 189,000 cells/cumm, creatinine level 0.9 mg/dL, alanine aminotransferase 70 U/L, aspartate aminotransferase 30 U/L, serum albumin 3.2 mg/dl, and total protein 11.4 mg/dl. Abdominal ultrasound showed minimal hepatomegaly with coarse echotexture and moderate splenomegaly measuring 17 cm in size. Six-month follow-up of the patient showed the patient was healthy and free of VL.

## 6. Discussion

Visceral leishmaniasis is one of the neglected tropical diseases the Nepalese government plans to eliminate. The efforts being used are active case surveillance, early diagnosis with ease of access to testing kits, integrated vector management, and the provision of standard treatment with LAMB. In addition to LAMB, other antileishmanial drugs are also available which can be used a single drug or in combination. The National Kala-azar Elimination Program includes LAMB (10 mg/kg single dose) as a first-line single-drug regimen for the treatment of the visceral leishmaniasis. Other combinations are also recommended as part of alternative second-line treatment. These combinations are LAMB (5 mg/kg) with miltefosine (50 mg BID for 7 days) or paromomycin (11 mg/kg base for 10 days) or combination of miltefosine with paromomycin for adults for 10 days. Third-line regimen includes infusion of plain amphotericin B (75–1 mg/kg daily or on alternate days for 15–20 dosages). Finally, fourth line details miltefosine for a total of 28 days.

The patient received 5 mg/kg of the total dose of liposomal amphotericin B as a treatment for the first episode within 3 days in the local hospital. He was diagnosed clinically and tested positive for the rK39 test. The efficacy of liposomal amphotericin B with this amount was found to be around 97% [4].

The patient became symptomatic after few months of treatment. He was diagnosed clinically with increasing splenomegaly and development of pancytopenia, and bone marrow aspiration showed LD bodies. He was treated with combination alternative therapy with LAMB 5 mg/kg first day followed by 15 mg/kg (11 mg/kg base) of paromomycin. The cure rate of this treatment per protocol has been found to be around 98.7% [3]. He was diagnosed with pulmonary tuberculosis with a cavity on the right lung. He was free from malaria and HIV. On follow-up, chest X-ray was clear.

The patient became symptomatic even after the second session of combination therapy. Despite showing improved laboratory parameters at the end of the treatment regimen of LAMB and paromomycin for the second episode, the patient had deteriorated further in the third episode as observed by the parameters. In a study published in 2011 (open-label, parallel-group, and randomised controlled trial), LAMB 5 mg/kg single dose followed by paromomycin 11 mg/kg for 10 days had shown a cure rate of 97.5% and 98.7% after six months on intention to treat and per protocol analysis, respectively. Even in this study, there were few patients who did not respond to the treatment [3]. The patient developed massive splenomegaly with severe pancytopenia and was diagnosed with VL on the basis of bone marrow aspiration which showed the presence of LD bodies. We evaluated for the comorbidities including tuberculosis, malaria, and HIV, which were negative. Four criteria for possible acquired haemophagocytic lymphohistiocytosis (fever, pancytopenia, splenomegaly, and high ferritin level) were positive; however, all tests could not be performed. Other causes for immunodeficiency could not be evaluated.

We started the patient on miltefosine monotherapy directly observed treatment. The patient started recovering with weight gain and improvement on blood parameter and negative bone marrow for parasite at the end of treatment. On further follow-up after six weeks following miltefosine treatment, patients showed good clinical and laboratory improvement. Six-month follow-up of the patient showed negative signs or symptoms of the disease.

Miltefosine has been shown to be ineffective with 20% relapse at one year and no more used as a first-line therapy in Nepal [5]. However, in our case, the end treatment cure was achieved as shown by the bone marrow report. This case has raised questions on available treatment efficacy for the treatment of VL. In conjunction with other studies of emerging resistance in the parasite, this case may be an indication for the concerned authorities to increase the active surveillance against VL so as to achieve the earlier goal of eliminating VL from Nepal [1, 6, 7].

The failure for treatment with standard care could be attributed to the emerging resistance against them. Hence, miltefosine directly observed treatment could still be one of the efficacious therapeutic alternatives in cases where the standard drug treatments fail to perform.
